# Exploring micronutrients and microbiome synergy: pioneering new paths in cancer therapy

**DOI:** 10.3389/fimmu.2024.1442788

**Published:** 2024-11-29

**Authors:** Kartik Bhatnagar, Kanupriya Jha, Nishu Dalal, Ninad Patki, Garima Gupta, Amit Kumar, Anil Kumar, Sarika Chaudhary

**Affiliations:** ^1^ Department of Biotechnology, School of Engineering and Applied Sciences, Bennett University, Greater Noida, Uttar Pradesh, India; ^2^ Gene Regulation Laboratory, National Institute of Immunology, New Delhi, India; ^3^ Azrieli Faculty of Medicine, Bar-Ilan University, Safed, Israel; ^4^ Biological Engineering and Sciences, Indian Institute of Technology Gandhinagar Palaj, Gandhinagar, Gujarat, India

**Keywords:** microbiome, cancer, micronutrients, probiotics, dysbiosis

## Abstract

The human microbiome is the complex ecosystem consisting of trillions of microorganisms that play a key role in developing the immune system and nutrient metabolism. Alterations in the gut microbiome have been linked to cancer initiation, progression, metastasis, and response to treatment. Accumulating evidence suggests that levels of vitamins and minerals influence the gut environment and may have implications for cancer risk and progression. Bifidobacterium has been reported to reduce the colorectal cancer risk by binding to free iron. Additionally, zinc ions have been shown to activate the immune cells and enhance the effectiveness of immunotherapy. Higher selenium levels have been associated with a reduced risk of several cancers, including colorectal cancer. In contrast, enhanced copper uptake has been implicated in promoting cancer progression, including colon cancer. The interaction between cancer and gut bacteria, as well as dysbiosis impact has been studied in animal models. The interplay between prebiotics, probiotics, synbiotics, postbiotics and gut bacteria in cancer offers the diverse physiological benefits. We also explored the particular probiotic formulations like VSL#3, Prohep, *Lactobacillus rhamnosus* GG (LGG), etc., for their ability to modulate immune responses and reduce tumor burden in preclinical models. Targeting the gut microbiome through antibiotics, bacteriophage, microbiome transplantation-based therapies will offer a new perspective in cancer research. Hence, to understand this interplay, we outline the importance of micronutrients with an emphasis on the immunomodulatory function of the microbiome and highlight the microbiome’s potential as a target for precision medicine in cancer treatment.

## 1 Introduction

The human microbiome comprises approximately 100 trillion microbial cells, with majority inhabiting the gut (1). The human gut microbiome refers to the total microbial population including archaea, fungi, viruses, and a complex hub of bacteria with diverse populations of Actinobacteria, Firmicutes, Proteobacteria, Bacteroides, Eubacteria, Bacteroidetes, Bifidobacteria, Cyanobacteria, Spirochaete, and many more (2–4). The makeup and diversity of the gut microbiome have been shown to influence numerous aspects of human health. Beyond the gastrointestinal (GI) tract, the gut microbiota has profound impact on immune surveillance, systemic inflammation, and metabolic pathways, all of which are linked to the initiation and progression of cancer (5). The gut microbiota influences the immune system and may interact with intratumoral microbiota, forming an immuno-oncology microbiome axis (6). Intestinal microbes interact with host cells to maintain intestinal barrier integrity and influence the bioavailability of therapeutic agents (7). The microbiome composition in cancer patients has been identified as a potential target for manipulation in both immunotherapy and cancer (8). Findings from the colitis-associated colorectal cancer (CAC) mice microbiota revealed the pivotal role in the predisposition of tumor development (9). The composition and diversity of the gut microbiome influence the cancer pathogenesis, progression, sustenance, and treatment outcomes. For example, a study found that greater diversity of gut flora in 55 cervical cancer patients undergoing chemotherapy and radiation therapy (CRT) was associated with better treatment outcomes. The outcome of the study suggested that benefited patients from CRT therapy have higher infiltration of activated CD4+ T cells and more diverse gut microbiome (10). High microbiome diversity in patients with rare long-term survival (LTS) for pancreatic ductal adenocarcinoma (PDAC) may reflect either the pancreas returns to healthy stasis or the host microbiome’s attempt to stifle unfavourable developments in the pancreas (11). In colorectal cancer (CRC) patients, the link between diet-microbiome interaction and composition supports the significance of the microbiome in cancer. A high-fat diet may alter the microbial composition, which in turn could influence cancer risk and prognosis. The negative correlations between the abundance of Bacteroides fragilis and the intake of dietary fats such as unprocessed pork (belly) and animal fats were studied. This suggests that higher consumption of these fats may be linked to lower levels of Bacteroides fragilis, potentially impacting cancer risk (12). Investigations in mouse models have revealed that bacterial strains such as *Lactobacillus casei* BL23 (13) and *Lactobacillus acidophilus* (14) can be used as probiotics to modify the microbiota composition for cancer treatment.

Micronutrients functions as anti-inflammatory and antioxidant agents through a variety of chemical mechanisms involving cell-signaling pathways. They exert their effects on cells by influencing epigenomic, transcriptomic, proteomic, and metabolomic alterations via gene expression modulation. Micronutrients also play a role in inhibiting cell proliferation, promoting apoptosis, and reduce angiogenesis by targeting the signaling pathways such as the PI3K/AKT/mTOR. Higher intake of micronutrients such as vitamin B12, vitamin D, folic acid, selenium, and antioxidants like carotenoids have been shown to significantly lower the risk of lung, CRC, prostate, and breast cancers (15, 16). Additionally, micronutrients are essential for the growth and function of gut microbes. Insufficient micronutrient intake can lead to dysbiosis by altering the composition and function of the gut microbiome (17, 18). These modifications may have an impact on immune response modulation, which is crucial in cancer development and progression (19). For example, patients with gastric cancer have been found to have lower blood levels of vitamin C, frequently in conjunction with *Helicobacter pylori* infection. Vitamin C is depleted as a result of *H. pylori* toxins interfering with its transportation inside the stomach lumen (20, 21). Supplementing with vitamin C has demonstrated promise in boosting immune responses and inhibiting tumor growth, underscoring its function in gut-host interactions that could impact the risk of cancer (22). Epidemiological studies present a positive correlation between micronutrient uptake and reduced cancer risk whereas the randomized controlled results concerning the effectiveness of dietary supplements in cancer prevention remain inconclusive. Moreover, the relationship between bacteria and tumors are intricate within the human body. Both direct and indirect degrees of interaction between gut microbiota and tumor have been investigated (23). *H. pylori* in GI malignancies and anaerobic bacterial species like Fusobacterium and *Bacteroides fragilis* in CRC are the two examples of direct connection (23–25). Indirect interactions between bacterial species have been observed in individuals with CRC and head and neck squamous cell carcinoma (HNSCC), respectively. Examples of these species are *Candida albicans* and *Fusobacterium nucleatum* (26, 27). Hence, outcomes to cancer disease such as survival and treatment response are affected by the gut microbiome with its diverse array of bacteria. By comprehensively understanding the mechanism through which different micronutrients interact with human gut flora and participate in cancer disease, researchers can develop new personalized therapies and treatment modalities for affected individuals. This review discusses the complex link between the gut microflora, micronutrients and cancer, showcasing the immense potential in harnessing microbiome-targeted strategies for enhancing cancer detection, prevention, and treatment to improve prognosis and patient longevity.

## 2 Gut microbiome and micronutrients in cancer

According to the statistical data from the International Agency for Research on Cancer (IARC), factors such as poor diet, lifestyle, smoking alongside overpopulation and aging, are expected to significantly escalate the worldwide burden of cancer by 2030 (28). The human gut microbiome not only affects the emergence of cancer (29) but also modifies the toxicity and efficacy of cancer therapies, including immunotherapy (30). The bacteria and fungus that make up the intratumor microbiome are involved in oncogenesis, tumor progression, and treatment response (31). Through a variety of processes, including DNA damage and alterations in gene expression and tumor microenvironment (TME), immune system, and host metabolism, microorganisms can either stimulate or inhibit cell growth and metastasis (32). Diet and nutrition play a key role in modulating the gut microbiome, impacting the bioavailability and metabolism of bioactive food constituents. Such interactions can eventually influence cancer development (33, 34). Vegetable-rich diets increase the abundance of fiber-degrading bacteria Firmicute and the synthesis of short-chain fatty acids (SCFAs) in the gut, impacting microbial functions (35). Differences in microbial functions were observed in 27 fecal samples from omnivorous, vegan, and vegetarian individuals, potentially impacting the development of colon cancer (36). With the improved cancer management in recent years, the uptake of micronutrient supplements like vitamins and minerals complements the standard therapy in patients (37). Research indicates that micronutrient deficiencies have an impact on nutritional status, immune function, and treatment tolerance in cancer patients. Malnutrition, particularly cachexia, is commonly observed among cancer patients and can lead to adverse treatment outcomes and reduced quality of life. Micronutrient supplementation, when appropriately selected and timed, can enhance patient compliance, reduce adverse effects, and improve treatment response, prognosis, and overall quality of life (38, 39). Studies have shown that micronutrients like selenium, vitamin D, and L-carnitine are crucial in supporting the immune functions and overall well-being of cancer patients.

### 2.1 Interactions between fat soluble vitamins, gut microbiome and cancer

#### 2.1.1 Vitamin A

**Figure 1:**
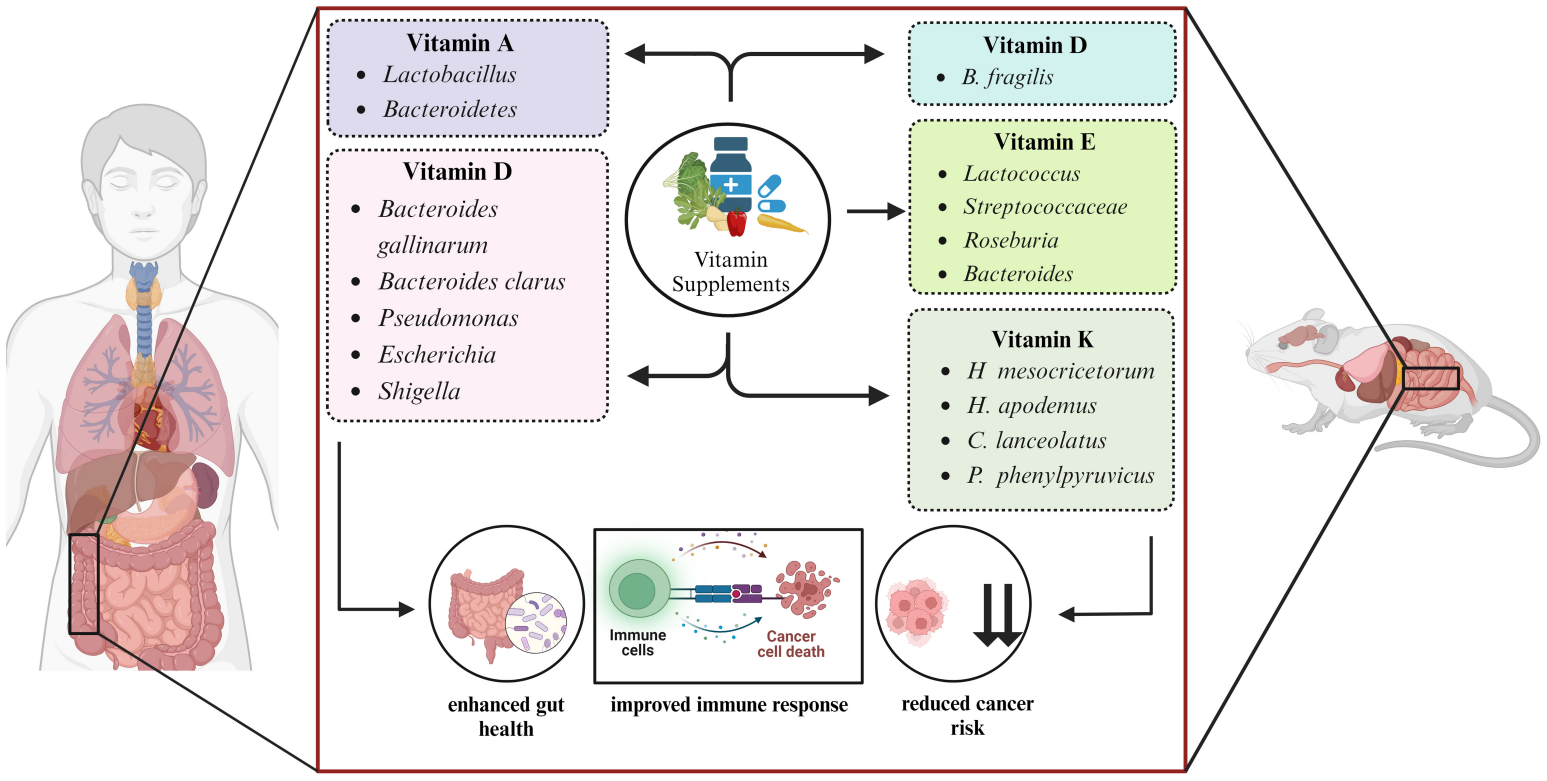
Microbe targeted vitamin supplementation modulates the host health.

The human GI tract provides a hospitable environment for a diverse array of microbes. These microbes play a crucial role in altering the essential nutrients needed for their survival (40). The relationship between gut microbiome, vitamin A metabolism, and host immunity plays an important role in maintaining health and preventing disease. Animal models and meta-analysis studies have demonstrated the protective effects of vitamin A and its derivatives against lung (41) and prostate cancer (42). Vitamin A homeostasis disruptions significantly impact immune cell migration and gut environment activity, critical for maintaining immunological balance (43–45). Studies have established a direct correlation between the gut microbiome composition and vitamin A plasma concentrations, suggesting the potential role of vitamin A in shaping the gut microbial community (46–48). A study in six-week-old CT26 induced BALB/c mice supplemented with βeta-carotene, a precursor of vitamin A, demonstrated the inhibition of cancer cachexia-related changes as well as restored the diversity of gut microbiome. The anti-cancer property of βeta-carotene involves systemic inflammation and lipolysis inhibition (49). In another study, vitamin A supplementation has shown potential in improving health outcomes by fostering the growth of beneficial microbes like Lactobacillus and Bacteroidetes (50–52). Early childhood vitamin A supplementation significantly impacts the development of the gut microbiota, promoting the maturation of the intestinal microbial ecosystem (53). This modulation of gut flora suggests the potential of vitamin A as an adjuvant therapy for infectious disease (**Figure 1**).

#### 2.1.2 Vitamin D

Emerging evidence suggests that vitamin D metabolism and functions are disrupted in numerous types of cancers, and it is crucial for treatment and prevention of cancer (54). Vitamin D directly controls tumor proliferation and differentiation while indirectly modulates the immune cells of TME, thereby demonstrating anti-cancer properties (55). In a study conducted on C57BL/6 mice, it was observed that increased availability of higher levels of vitamin D provides a greater display of immune-dependent resistance to cancer. In mice, vitamin D activity on intestinal cell lining is responsible for resistance as it modifies the microbiome composition in favor of *Bacteroides fragilis* and enhances the immune response against cancer (56). As studied in 74 CRC patients, vitamin D supplementation enhances the diversity of *Bacteroides gallinarum*, and *Bacteroides clarus* and shapes the gut microbiome (57). A high dosage of vitamin D3 treatment significantly decrease the prevalence of common opportunistic pathogen species, including *Pseudomonas, Escherichia*, and *Shigella*, leading to an increase in phylotype richness in human gut microbiome (58) (**Figure 1**).

#### 2.1.3 Vitamin E

Lipid-soluble vitamin E contains eight naturally occurring isoforms: α, β, δ, and γ isoforms of tocopherol and α, β, δ, and γ isoforms of tocotrienol, contributing to the anti-cancerous effect of vitamin E (59). Reduced levels of vitamin E have been linked with a higher cancer risk. However, supplementation of α-tocopherol has a favorable effect on lowering cancer risk (60). In mice, tocotrienol has been demonstrated to modify the gut microbiome composition, possibly restoring gut health to a more favorable state. Mice infected with colon cancer when supplemented with δ-tocotrienol exhibit increased levels of *Streptococcaceae*, *Bacteroides*, and *Lactococcus* bacteria. This therapeutic effect of tocotrienol exerts anti-inflammatory action and provides benefit to the gut flora (61). In mice, the enhanced diversity of gut microbiota including Roseburia, Bacteroides and Lactococcus, was observed when δ-tocotrienol in conjunction with δTE-13 metabolite, was administered and demonstrated a beneficial impact in preventing colon cancer and reducing colitis in mice (62)​ (**Figure 1**).

#### 2.1.4 Vitamin K

Vitamin K acts as a coenzyme and catalyzes the carboxylation reaction of vitamin K-dependent proteins (63). Vitamin K1 (phylloquinone) and vitamin K2 (menaquinone) are essential cofactors for γ-carboxylation and alterations in γ-carboxylation levels have significant implications in cancer development and progression (64). Anti-inflammatory and antioxidant effects of vitamin K prevents cellular senescence and has a potential anticancer effect in pancreatic and prostate cancer (65). A study in human colorectal cell line Caco-2 and mouse macrophage cell line RAW 264.7 tested the 86 different gut bacteria strains. The outcome of this study demonstrated the crucial role of vitamin K2 in inhibiting the secretion of pro-inflammatory cytokines like interleukin 8 (IL-8) in Caco-2 cells while interleukin 6 (IL-6) and tumor necrosis factor-α (TNF-α) in RAW 264.7 cells (66). In mice induced with azoxymethane (AOM), vitamin K2 administration lowered the susceptibility to colon carcinogenesis and decreased the presence of CRC-causing microbes such as *H. apodemus* and *H. mesocricetorum*, while an increase in certain species such as *Curvibacter lanceolatus, Psychrobacter phenylpyruvicus*, and *Parasutterella excrementihominis* were observed (67) (**Figure 1**).

### 2.2 Interactions between water soluble vitamins, gut microbiome and cancer

#### 2.2.1 Vitamin B

Vitamin B complex plays a multifaceted role and helps in regulating immune cell activity, bacteria survival, and overall gut microbiome health. B Vitamins exhibit prebiotic potential, influencing the gut microbiome composition. Bifidobacterium and Lactobacillus species within the gut microbiome have been reported to synthesize several B vitamins (68, 69). Increased vitamin B2 concentrations in the blood and diet decrease the CRC risk as reported in a meta-analysis (70). Interestingly, thiamine supplementation has been shown to inhibit tumorigenesis in certain cancer types (71) and plays a role in developing immunotherapies for cancer eradication. A study in patient tissue samples infected with *H. pylori* showed lower levels of riboflavin plasma concentrations compared to the non-infected group. Thus, *H. pylori* was validated to be associated with riboflavin absorption and upregulation of stomach cancer in patients (72). Commensal bacteria lower the risk of CRC by producing the vitamin B2 precursor 5-OP-RU, which also enhances the intestinal mucosa’s defense against infections through immunological mechanisms (73, 74). Vitamin B5 originating from the gut microbiome stimulates the formation of Tc22 immune cells that produce interleukin 22 (IL-22) and have potent anticancer effects and strong immunotherapy responses (75). This diverse immunological and anti-cancer effects of B vitamins collectively highlight the potential therapeutic value of commensal bacterial strains capable of synthesizing these essential micronutrients (76).

#### 2.2.2 Vitamin C

Vitamin C exhibits potent anti-tumor properties and directly induce apoptosis through the production of hydroxyl radicals via the Fenton reaction. The pro-oxidant properties of vitamin C contribute to tumor growth inhibition by regulating the gene expression (77). Vitamin C intake has been linked to reduce the risk of developing gastric cancer (78), bladder cancer (79), breast cancer (80), glioma (81), cervical tumors (82), prostate cancer (83), pancreatic cancer (84) and overall cancer incidence. It has been demonstrated that vitamin C can prevent hypoxia and oncogenic kinase signaling, reverse the epithelial-to-mesenchymal transition (EMT), and enhance the immune system (85). With its anti-microbial properties, vitamin C modulates the microbial diversity in the intestine (86). According to a study in human gastric biopsy specimens and six-week-old Mongolian gerbils inoculated with *H. pylori*, vitamin C inhibited the growth of 64 *H. pylori* strains isolated from human biopsy samples and similar inhibitory effect was also observed in an *in vivo* model after oral administration of vitamin C (87).

### 2.3 Role of essential minerals in gut microbiome and cancer

Microelements such as iron, zinc, and selenium play crucial roles in tissue functioning and cellular metabolism. Microbes constitute 200 grams of the total body mass of a healthy individual (88). Interactions between microelements and gut microbiome disturb the microbial niche, affecting gut health. The function and configuration of the gut flora are amended by the shortage or excess of mineral composition (89). The gut microbes have evolved the mechanism of transportation and binding microminerals with high affinity and it is crucial as the levels of bioavailable microminerals necessary for their growth are typically low (90). Studies in 30 male C57BL/6 mice fed with magnesium-deficient diet for six weeks reported a change in the microbial composition of the gut (91).

#### 2.3.1 Iron

**Figure 2:**
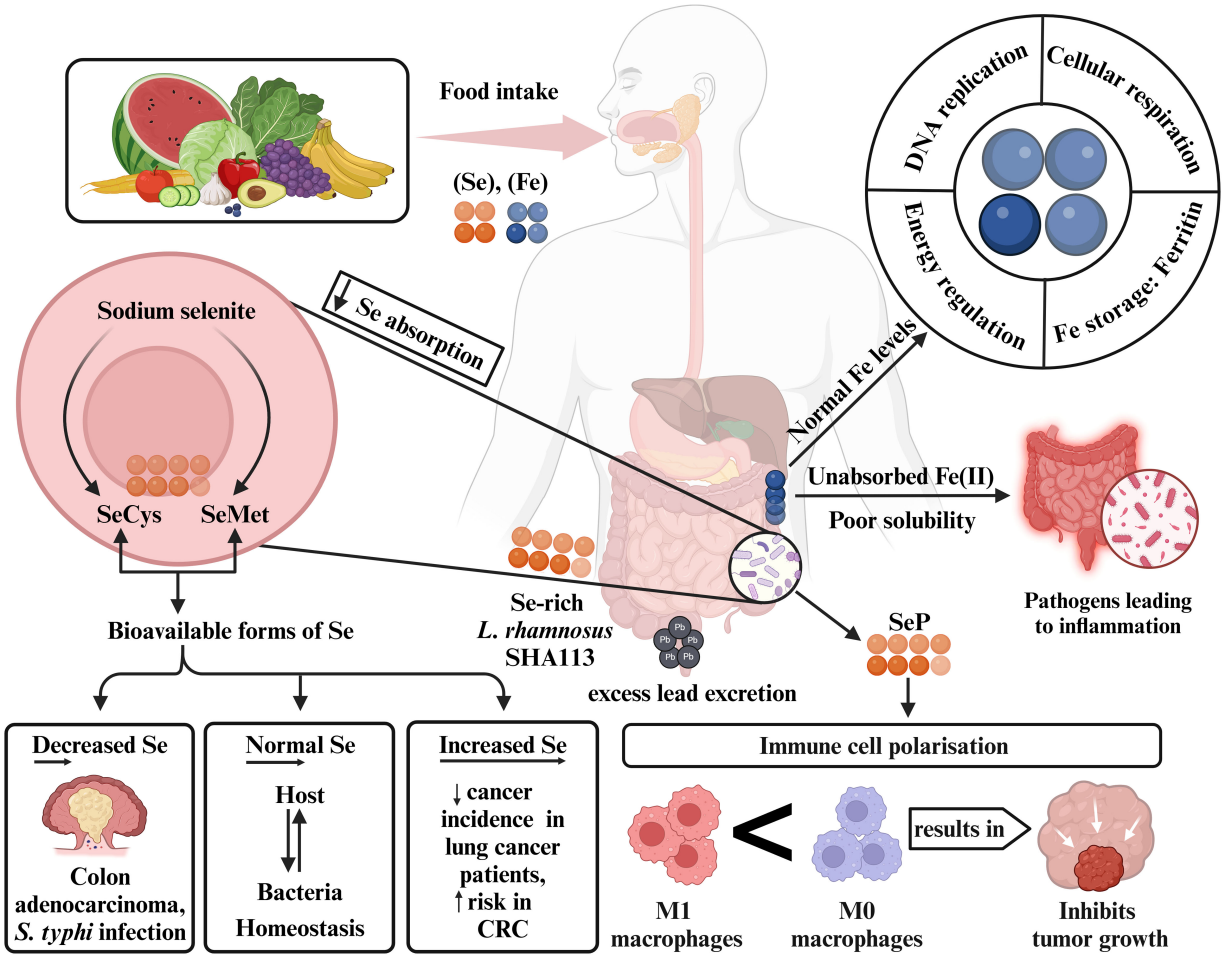
Modulation of gut microbiota based on Se and Fe levels. Adequate dietary intake supports Se and Fe biosynthesis. Low Se absorption by gut microbes leads to sodium selenite metabolizing into SeCys and SeMet. Deficient Se levels may increase colon adenocarcinoma and *S. typhi* infection risk, while elevated levels are linked to CRC. Normal Se levels promote homeostasis between host and microbiota, aided by *Lactobacillus rhamnosus*, which helps prevent colon issues by eliminating excess lead. SeP present in gut microbial genes influence immune cell polarization, reducing tumor progression. Unabsorbed Fe(II) can trigger gut inflammation, but normal Fe levels support essential functions like DNA replication, energy regulation, and cellular respiration. Se, selenium; Fe, iron; SeCys, selenocysteine; SeMet, selenomethionine; SeP, selenoproteins; Pb, lead; CRC, colorectal cancer.

Iron (Fe) is required as a trace element for many different biological functions, such as energy consumption, cellular respiration, DNA replication, and iron-dependent signaling (92–94). Further, it plays a crucial role in cancer biology, especially in cancer stem cells (CSCs) and mesenchymal cancer cells (95). As reported by Andrews et. al, bacteria need 10^-7^ – 10^-5^ M of iron for optimal growth (96). Due to the poor solubility, iron remains unabsorbed in the colon and thereby promoting the proliferation of intestinal pathogens (97, 98). A study in 6–14-year-old African children stated that microbes such as Lactobacillus and Bifidobacterium require iron in very minute quantities for survival, whereas *E. coli*, Shigella, and Salmonella depend on iron for colonization and to enhance their virulence (99). Consumption of iron-enriched diets is associated with the higher levels of Proteobacteria which, in turn, reduces the microbial activity (100). However, due to the distinctive iron withholding mechanisms, the availability of iron to microorganisms is restricted and hence pathogen growth is inhibited (101). At the site of the large intestine, Bifidobacteriaceae family bacteria bind to the iron limiting the formulation of free radicals and reducing the risk of CRC (102). The growth of Bifidobacterium is promoted by the globular protein Lactoferrin that shows a high affinity towards iron and has bifidogenic properties (103) (**Figure 2**).

#### 2.3.2 Zinc

**Figure 3:**
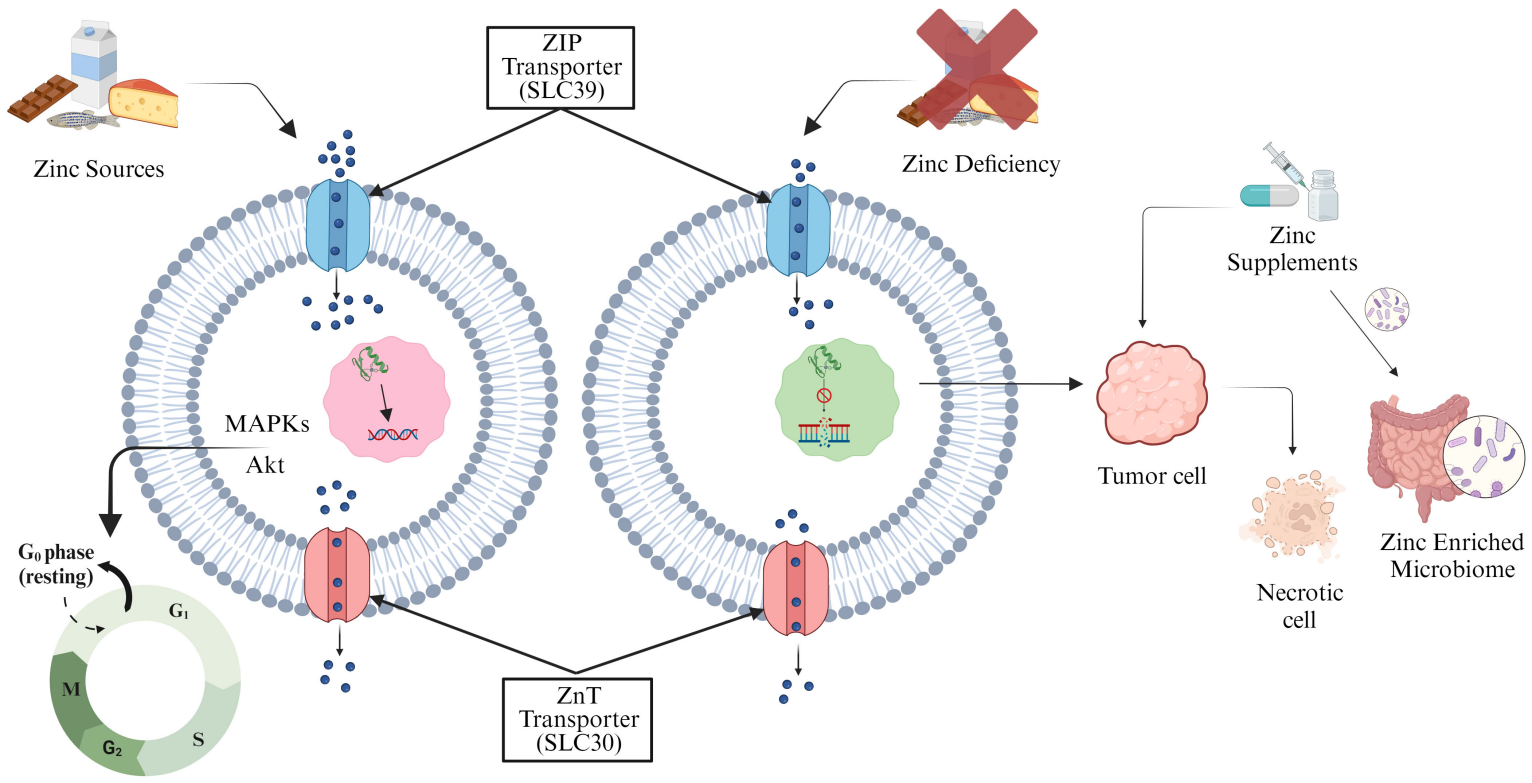
Zinc transporter proteins regulate cellular Zn storage and distribution. Adequate Zn levels maintain controlled phosphorylation-dependent MAPKs and Akt cell cycle signaling pathways (left). Zn deficiency induces DNA damage and cancer. Zn supplementation triggers necrotic response in tumor cells, inhibiting division, and enhances gut microflora, offering additional benefits (right).

Zinc (Zn), an essential micronutrient required for several biological functions including DNA binding and synaptic transmission, is found in more than a thousand DNA-binding proteins (104). It is necessary for the action of Zn-containing DNA-repair enzymes, which are vital for mending damage to DNA. Lack of Zn can impair these enzymes capacity to fix damaged DNA, which can accelerate the onset of cancer (105). The metal transporter families, ZIP (SLC39) and ZnT (SLC30) are involved in maintaining Zn homeostasis, where ZIP increases the cytoplasmic Zn concentrations and the ZnT reduces the concentration (106, 107). Research conducted on Zn-deficient N-nitrosomethylbenzylamine (NMBA) rats revealed a higher frequency of esophageal cancer in humans. The development of endophytic tumor is permitted by the attenuated form of NMBA carcinogen in conjunction with a Zn shortage (108–110). Zn deficiency decreases the microbial population in the gut and results in the modification of SCFAs output (111). Bacterial species such as *Haemophilus influenzae*, *E. coli*, *Campylobacter jejuni*, and others require Zn for their virulence and colonization (112). A study in Indonesian preschool children reported that taking Zn supplements for 90 days along with the probiotic *Lactobacillus plantarum* greatly boosts the humoral immune response (113). Increased immunotherapy efficacy occurs through the activation of immune cells including dendritic and CD8 T cells, facilitated by the delivery of by Zn ions delivered into the TME. Mice with hepatocellular cancer (HCC) have been used to illustrate this (114). Zn supplementation causes a potent necrotic response in tumor cells and activates specific signaling pathways in prostate cancer, including ERK1/2 and protein kinase C (PKC), and prevents the division of tumor cells (115). The proliferation of bacteria, including *Prevotella*, *Proteobacteria*, and *Actinobacteria*, has been linked to zinc enrichment of the microbiome and benefit cancer therapy. The ability of Zn to regulate the gut epithelial wall helps in the regeneration of impaired intestinal epithelium (116, 117) (**Figure 3**).

#### 2.3.3 Selenium

Selenium (Se), an important trace element of the human body plays a major role in cancer prevention through its essential chemical compounds, L-selenomethionine (SeMet), sodium selenite, and selenocysteine (SeCys) (118, 119). The low absorption of inorganic Se in human cells is typically caused by lactic acid-producing bacteria that convert sodium selenite into SeCys and SeMet, thus facilitating the production of bioavailable forms of Se (120). Lactobacillus increases the Se concentrations in human cells as reported in thyroid adenomas (121). Bacteria such as Enterobacteriaceae*, C. difficile, and E. coli.* are known to colonize the human gut because of the selenoproteins encoding genes (122). Findings from clinical trials and epidemiological studies on lung cancer patients suggest that increased Se levels reduce the risk of cancer recurrence and incidence (123). Elevated levels of selenoprotein P were adversely correlated with CRC risk, although lower expression of several selenoproteins was observed in colorectal adenoma and cancer tissues (124). Se-enriched yeast supplementation has shown encouraging outcomes in decreasing the occurrence and mortality of colorectal, prostate, and lung cancers (125). Se-rich *Lactobacillus rhamnosus* SHA113 aids in lead excretion through stools and prevents lead damage to the gut (126). Minimal Se consumption results to a gut microbiome pattern that is more susceptible to *S. typhi* infections (127). Se administration alters immune cell polarization within the TME, promoting the antitumor pro-inflammatory and anticancer M1 macrophage phenotype over the immunosuppressive M2 phenotype. Additionally, it increases the activity of neutrophils, natural killer (NK) cells, and CD8+ T lymphocytes, promoting antitumor immunity and potentially inhibiting tumor growth and progression (128, 129). A study in eight-week-old mice supplemented with Se showed an increase in the number of microbes belonging to the Lactobacillus genus along with Ruminococcaceae, Christensenellaceae, and Lachnospiraceae niche (130) (**Figure 2**).

#### 2.3.4 Copper

The biochemistry of human beings depends heavily on the trace element copper (Cu) (131). In human body Cu is required as a micronutrient for several physiological processes, including oxygen transportation, enzyme functioning, and cell signaling (132). Cu plays a crucial role in cell proliferation and cancer progression, as evidenced by increased Cu levels in cancer cell lines, tissues, and patient serum (133–136). Cu-binding proteins, such as mitogen activated protein kinase 1 (MEK1) and UNC-51-like kinases 1 and 2 (ULK1/2), facilitate signal cascade activation, promoting tumorigenesis (137). Cu is an essential regulator of the autophagic kinases ULK1/2 to drive lung adenocarcinoma. Cu chelators such as tetrathiomolybdate (TTM) and Cu_4_ (2,4-di-tert-butyl-6-(1H-imidazo- [1, 10] phenanthrolin-2-yl)phenol)_4_]·10 CH_3_CN, a bioinorganic complex 2, have been proposed as potential cancer drugs due to their ability to inhibit Cu-dependent signaling pathways (138). Inflammatory cytokines, which mobilize Cu, are linked to Six-Transmembrane Epithelial Antigen of the Prostate 4 (STEAP4) activation, enhancing Cu uptake and X-Linked Inhibitory Apoptosis Protein (XIAP) activation, thereby suppressing apoptosis in colon cancer (139). Animal study on pigs shows that the excess of Zn and Cu leads to stability of the intestinal microbiome and provides resistance to the antimicrobial agents, and antibiotics (140). Shao et al. in his study observed that people living in metal-polluted regions have alterations in their gut microbiome due to long-term exposure to multiple metals including Cu (141). Toxic metals, including Cu, have a detrimental effect on the gut microbiota and an increase in its concentrations leads to the reduction of the diverse bacterial community (142).

## 3 The tripartite microbiome: bacterial, viral, and fungal interactions from gut to tumor

### 3.1 Bacterial influence in gut and tumor microenvironments

#### 3.1.1 Fusobacterium nucleatum

In CRC, Fusobacterium species, particularly *F. nucleatum*, are observed to be more abundant as compared to healthy or precancerous colon tissues (29, 143, 144). Originating from the oral cavity, *F. nucleatum* is detected in over 40% of CRC patients’ oral samples, suggesting a link between oral and gut microbial shifts in CRC progression (145). In esophageal cancer tissues, the DNA levels of *F. nucleatum* was detected in 23% of cases, suggesting a potential association with cancer development. In patients positive for *F. nucleatum*, a higher cancer-specific mortality rate was observed with the bacterium linked to the activation of chemokines, particularly C-C motif chemokine ligand 20 (CCL20), which is involved in immune cell migration and tumor progression (146). A notable dominance of *F. nucleatum* subsp. *animalis* has been reported in CRC tissues. Microbiome analysis of human tumor tissue and metagenomic analysis of stool samples consistently indicates a clear enrichment of *F. nucelatum animalis clade 2*, especially in the proximal colon, with its presence varying across patients tissues from approximately 10% to 90% (147, 148). This observation has been validated across diverse geographic regions, sequencing cores, age groups, and computational approaches, all contributing to the same outcome that CRC creates a unique microenvironment that fosters Fusobacterium colonization. Using metagenomic sequencing, it was revealed that colorectal tumor may facilitate an environment that promotes Fusobacterium proliferation, potentially driving colorectal liver metastases (149). Transcript levels of *F. nucleatum* were found to be up to 400 times higher in CRC tumor tissues as compared to the healthy tissues, further underscoring its role in cancer development (144, 150, 151). Moreover, the association between *F. nucleatum* and liver metastasis expands the potential role of *F. nucleatum* in cancer (149). In mouse models, *F. nucleatum* reduces gut microbiota diversity and linked to the development of colorectal cancer liver metastasis (CRLM). This dysbiosis influences the immune response in the liver, further promoting cancer growth (152). Mechanistically, *F. nucleatum* connects with the host endothelial and epithelial cells through FadA adhesion protein, allowing internalization into host cells. This interaction triggers proinflammatory signaling pathways, including nuclear factor (NF)-kB and IL-6, promoting a microenvironment conducive to tumor growth (153, 154). *In vitro* studies have shown that outer membrane vesicle (OMV) secreted by *F. nucleatum* stimulate intestinal epidermal cells to produce IL-8 and TNF-α, initiating inflammatory response that further support tumorigenesis (155). Additionally, *F. nucleatum* localizes at tumor sites by binding through its Fap2 lectin to carbohydrate moiety present on the tumor surface in CRC (156) and breast cancer (157). This interaction potentially facilitates EMT, a process associated with cancer cell invasion, metastasizing, stemness, and therapy resistance (158).

#### 3.1.2 Helicobacter pylori

*H. pylori* infection affects over 50% of the global population and is strongly linked to various GI diseases, including gastric cancers, peptic ulcers, and mucosa-associated lymphoid tissue (MALT) lymphomas (159–161). During *H. pylori* infection, MUC1, a transmembrane mucin glycoprotein, is upregulated and undergoes shedding (162). In this process, MUC1 exhibits altered glycosylation and impacts its protective barrier function (163). In gastric cancer, the change in function of MUC1 role from anti-inflammatory to pro-inflammatory contributes to chronic inflammation and cancer development (164). *H. pylori* recruit the polymorphonuclear neutrophils (PMNs) and indirectly alters the cancer progression, as outlined by the Correa pathway (165, 166). This pathway promotes a Th1-like immune response facilitated by pro-inflammatory cytokines such as interferon-γ (IFN-γ), interleukin-1β (IL-1β), and TNF-α, which further exacerbate inflammation and cancer development (167–169). The translocation of *H. pylori* into the host gastric epithelial cells is mediated by interactions between HopQ protein of the bacteria and host carcinoembryonic antigen-related cell adhesive molecules (CEACAM) (170, 171). Once inside the host epithelial cells, *H. pylori* deliver its cytotoxin-associated gene A (CagA) protein via a type 4 secretion system (T4SS), directly linking this mechanism to the development of gastric cancer (172). The injection of CagA leads to aberrant cellular signaling, including interactions with Src homology 2 (SH2) domain-containing protein tyrosine phosphatase 2 (SHP2) and phosphoinositide 3-kinase (PI3K), driving the transformation of normal epithelial cells into neoplastic ones (173).

#### 3.1.3 Bacteroides fragilis

*B. fragilis* is Gram-negative anaerobic symbiont that constitutes about 25% of anaerobes in human colon. The enterotoxigenic *B. fragilis* (ETBF) strain is closely associated with the induction of colitis and colon tumorigenesis, as evidenced in both colorectal neoplasia patients and fecal samples of CRC patients (24, 174). Moreover, the ETBF strain is considered as an ‘alpha bug’ due to its direct involvement in cancer development, capability to manipulate microbial communities and its unique virulence factors that promote tumorigenesis (175). In murine model study, ductal colonization with ETBF significantly accelerates breast tumor development and metastatic progression (176). In CRC, the ETBF-encoded metalloprotease *Bacteroides fragilis* Toxin (BFT) results in tissue impairment and chronic intestinal inflammation by activating critical signaling pathways, including Wnt/β-catenin and NF-kB (177–179). This inflammation caused by ETBF is a driver of CRC, as the infection significantly alters the regulation of cellular ‘stemness.’ Additionally, BFT-positive *B. fragilis* contributes to localized bacterial dysbiosis by encouraging the proliferation of other cancer-promoting bacteria. This imbalance disrupts the host immune system and foster a pro-inflammatory environment conducive to CRC development (180). Through a toll-like receptor 4 (TLR4)-dependent pathway, ETBF increases levels of transcriptional and epigenetic regulators, which promote tumor growth both *in vivo* and *in vitro* (181). The resulting pro-inflammatory and tumorigenic environment in the gut highlights the dual role of *B. fragilis* in both normal gut symbiosis and cancer promotion within the TME.

#### 3.1.4 Escherichia coli

The B2 and D phylogroups of *E. coli* are implicated in both intestinal and extra-intestinal diseases, contributing significantly to cancer initiation within the gut-tumor axis. These *E. coli* strains trigger cancer initiation and manipulation of host cell cycle by inducing inflammation, oxidative stress, and alteration in the cellular niche (182). Enteroaggregative *E. coli* (EAEC) is a significant intestinal pathogen that causes inflammation in the intestine (183). When EAEC binds to its intestinal receptor, MUC1, it enhances bacterial adhesion by interacting with adherence fimbriae (AAF) (184). The development of CRC has been largely attributed to the diversity of *E. coli*, particularly in individuals with mucosa-internalized and mucosa-associated CRC (185, 186). Additionally, eukaryotic epithelial cells are damaged by interstrand crosslinks and double-strand DNA breaks induced by colibactin, a protein secreted by pathogenic strains of *E. coli* and encoded by the pKs pathogenicity island (187–189). In CRC, *E. coli* enhances tumor growth by promoting cellular senescence and the secretion of growth factors that facilitate proliferation as demonstrated in both CRC mouse models (190, 191) and CRC human biopsies (191). *E. coli*-induced DNA damage responses are consistently observed in CRC, with a notable impact on Wnt signaling pathways, further driving cellular transformation and tumor growth (187, 191–193). Likewise, *E. coli* infection in the urinary bladder promotes chronic inflammation and activates the NF-κB pathway (194). This activation, driven by bacterial lipopolysaccharide (LPS), inhibits apoptosis and increases inflammation (195, 196).

#### 3.1.5 Propionobacterium acnes

*Propionobacterium acnes*, is a Gram-positive bacterium that is commonly found in the sebaceous (oil) glands of the skin (197). The gut microbiome influences systemic inflammation and immune responses and affect skin conditions like acne. The gut-brain-axis suggests that stress and emotional factors can alter gut microbiota further impacting the skin health (198). *P. acnes* has been reported in prostate cancer in numerous investigations (199, 200). *P. acnes* strains isolated from prostate cancer tissues exhibited inflammatory response, characterized by elevated secretion of IL-6 and IL-8 when cocultured with RWPE1 prostate epithelial cells. This suggests that *P. acnes* play a role in promoting chronic inflammation within the TME (199). Similarly, Cohen et al. observed a substantial change in the surface characteristics of *P. acnes* strains present in prostatic tissues when compared with isolated skin samples, highlighting carcinogenic infection of *P. acnes* with a higher degree of prostatic inflammation (200). In an *in vivo* induced mouse model of prostate cancer, *P. acnes* infection further highlighted its role in exacerbating inflammation and promoting cell proliferation, contributing to tumor development (201).

### 3.2 Viral contributions to gut dysbiosis and tumor development

Apart from bacteria, a considerable number of viruses also coexist in the human gut and are collectively known as the gut virome. Single-stranded and double-stranded DNA and RNA viruses compose the healthy adult gut virome (202). The human virome is primarily composed of phages that can infect and lyse bacteria in the gut, and Myoviridae, Podoviridae, Siphoviridae, and Microviridae are present dominantly. Viruses that infect eukaryotic cells constitute less than 10% of the total virome volume (203). Additionally, Hannigan and colleagues studied individuals with healthy colons, adenomas, and CRC. They found that bacteriophages, belonging to the Siphoviridae and Myoviridae families, were more common in people with CRC. These findings suggest that the types of viruses present in the gut are linked to CRC and may contribute to the disease by affecting the bacterial populations (204). Researchers compared the viromes of fecal samples from CRC patients and non-CRC controls. They identified 22 viral genera that distinguished CRC patients from healthy control. Additionally, they found that the gut bacteriophage diversity was higher in CRC patients than in controls (205). In contrast, another research group observed a decreased virome diversity in a mouse model of colorectal neoplasia. However, this alteration occurred after the appearance of the first tumor. These findings suggest a correlation between tumor development and gut virome alteration. At the genus level, Brunovirus and Hpunavirus were positively correlated with tumor growth (206). Earlier studies of the virome faced several limitations, including low sensitivity of sequencing technologies, difficulty in establishing a causal role of the virome in disease pathogenesis, and lack of entire viral genome annotations (207). Genome or gene clustering approaches appear to be promising avenues for elucidating the phylogeny of newly discovered gut viruses (208).

### 3.3 Fungal microbiota: implications for gut health and tumor growth

Fungi have emerged as key players in the diagnosis and progression of cancer. A study by Liu et al. revealed a significant increase in the abundance of 93 out of 108 fungal species, such as *Candida pseudohaemulonis*, *Aspergillus ochraceoroseus*, *A. rambellii*, and *Malassezia globose*, in CRC patients compared to healthy individuals. These findings suggest that complex interactions between bacteria and fungi, including the upregulation of D-arginine and D-ornithine and the stimulation of the butanoate metabolism pathway, may contribute to the development and progression of CRC (209). Moreover, qPCR analysis targeting the fungal 5.8S ribosomal gene revealed elevated fungal loads in diverse tumor types, including breast, melanoma, lung, colon, ovary, pancreas, brain, and bone, compared to the respective negative controls. Notably, fungal load varied across the above-mentioned tumor types. Subsequent internal transcribed spacer 2 (ITS-2) amplicon sequencing confirmed increased fungal abundance in these cancers. Interestingly, statistical analysis revealed that five fungal taxa—*Malasseziomycetes*, *Saccharomycetes*, *Diothideomycetes*, *Sordariomycetes*, and *Candida* were significantly overrepresented across these cancer types (210). Furthermore, the PDAC mycobiome was found to be distinct from both the gut and normal pancreatic mycobiomes, with a significant enrichment of Malassezia species in both mice and humans. Experimental studies involving fungal adoptive transfer and ablation suggest that specific fungal species can promote PDAC progression. This mechanism involves oncogenic Kras-induced inflammation leading to fungal dysbiosis, which subsequently activates the mannose-binding lectin C3 (MBL-C3) cascade and accelerates tumor growth (211). Similarly, abundance of 15 fungal biomarkers was significantly elevated in gastric cancer patients compared to a control group. Specifically, *Candida albicans* and *Alternaria* species were enriched. Given its association with gastric cancer, *C. albicans* could potentially serve as a fungal marker for this disease. Zhong et al. hypothesized that *C. albicans* may contribute to gastric cancer pathogenesis by reducing fungal diversity (212). Therefore, the emerging field of mycobiome research holds significant promise for understanding the role of fungi in human health and disease. Consequently, future research may uncover novel fungal biomarkers and therapeutic targets to combat cancer.

## 4 Microbial dysbiosis in cancer pathogenesis

Dysbiosis is characterized by an altered composition of gut microbiome, which disrupts the physiological homeostasis within intestinal epithelial cells (213). Dietary changes, antibiotic use, and inflammatory bowel disease contribute to dysbiosis (214). Disruptions in gut microbial activity hinder the continuous synthesis of microbial primary metabolites such as amino acids, vitamins, and polysaccharides (215). Secondary metabolites such as alkaloids, phenols, antibiotics, and others are highly crucial for defining the function and specificity of the diverse gut microbiome. The microbiota alters the TME and immune responses, impacting the effectiveness of cancer therapies and contributing to treatment-induced dysbiosis (216). The imbalance in the gut microbiome reduces the number of beneficial bacteria while increasing the number of cancer-causing bacteria. Additionally, gut microbiota dysbiosis can lead to increased inflammation and cholestasis in the liver, further promoting the development of pancreatic and HCC cancer (217). As per study conducted in fecal samples of 61 lung cancer patients, the loss of microflora diversity was observed when compared with the healthy stool samples of 28 individuals. There was a significant decrease in anti-inflammatory and SCFAs-producing bacteria and an abundance of tumor-promoting bacteria in these patients (218). Gut microbial metabolites, differing significantly in lung cancer patients, may serve as biomarkers for non-invasive diagnosis and enhance immunotherapy efficacy by influencing lung immune status through the gut-lung axis (219). Similarly, another study unveiled the association between dysbiosis and cholangiocarcinoma (CCA) development where microbial taxa such as Bifidobacteriaceae, Enterobacteriaceae, and Enterococcaceae were linked. It was observed that abnormal microbiome diversity fuels the disease progression via creating a feedback loop and accumulation of genetic and epigenetic changes (220). In elderly patients with HCC, the levels of Firmicutes, Actinobacteria, and Synergites were low as compared to the normal group where the Proteobacteria, Fusobacteria, and Tenericutes were in abundance. According to Yang et al., the fecal microbiome diversity of Fusobacterium and Flavonifractor was linked to young-onset colorectal cancer (yCRC), whereas the abundance of Streptococcus was observed in old onset colorectal cancer (oCRC). The dominance of *Flavonifractor plautii* in yCRC and *F. nucleatum* as a crucial microbiome in both yCRC and oCRC was confirmed with the sequencing methods. The findings from the study concluded that CRC is more closely related to young people because of the connection between diet and lifestyle factors (221). The gut microbiota composition between the CRC patients and healthy patients revealed the dominance of Bacteroides and Prevotella group in cancer patients compared to normal individuals. In contrast, there were no alterations in other common gut bacteria, including *C. leptum, C. coccoides*, *Lactobacillus/Leuconostoc/Pediococcus*, *Bifidobacterium*, *E. coli*, and *Faecalibacterium prausnitzii.* Likewise, qPCR analysis of stool samples revealed a higher amount of genetic material from bacteroides in colon cancer patients (222). Research has demonstrated substantial differences in the microbiota of individuals with gastritis, intestinal metaplasia (IM), and gastric cancer, suggesting a potential involvement of dysbiosis in disease progression. The result from this study reveals the enrichment of pro-inflammatory oral bacterial species, lactic acid-producing bacteria, and SCFAs synthesis pathways as relevant mechanisms contributing to gastric cancer (223). To delve deeper into the potential role of microbiome led dysbiosis in distinguishing between tumor patients and healthy patients, further investigation is required with an emphasis on clinical validations and elucidation of underlying mechanisms.

## 5 Tweak to treat: biotics and microbiome crosstalk in cancer

### 5.1 Prebiotics

**Figure 4:**
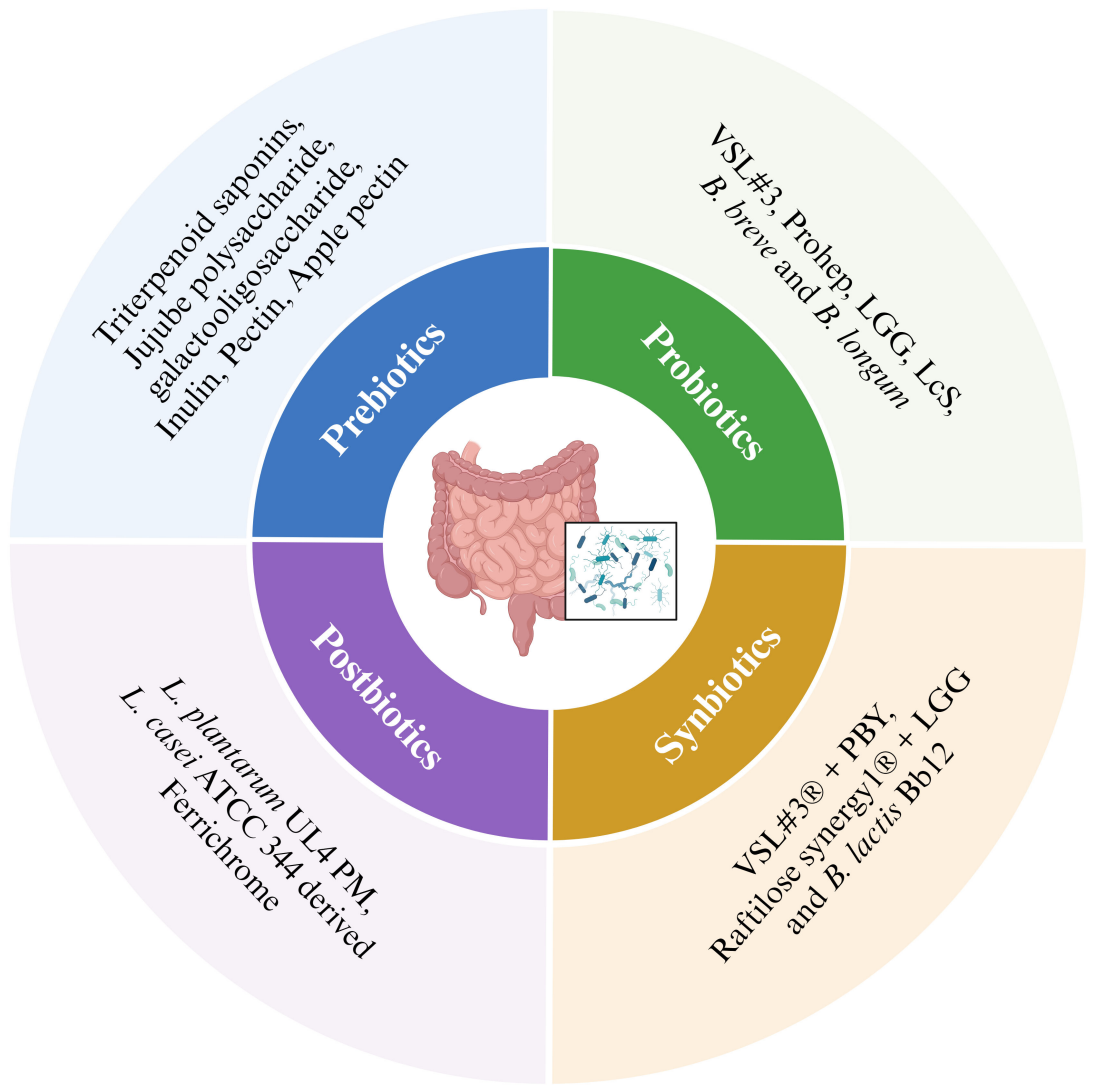
Microbes and biotics in cancer treatment.

Prebiotics are characterized as selectively fermented non-digestable dietary fibers that support the growth of probiotic microorganisms and their role in maintaining the intestinal microbial homeostasis and alleviating gut dysbiosis contributes to overall host health. Prebiotics act in colon and modulate the levels of resident Lactobacilli and Bifidobacteria to produce SCFAs (224). These synthesized SCFAs regulate a range of gut and ex-gut functions, including gut epithelial and mucus barrier, immune function, glucose and lipid metabolism, energy expenditure, and satiety (224, 225). Majority of the prebiotics are derivatives of carbohydrate group and possess the ability to withstand stomach enzymes (226). Several prebiotics such as triterpenoid saponins from *Gynostemma pentaphylum* (GpS) lower the colon polyps. Likewise, jujube polysaccharide, galacto-oligosaccharides, inulin and Djulis (*Chenopodium formosanum*) exhibited prevention of CRC development (**Figure 4**) (227). Upon administration with a complex prebiotic composed of four different oligosaccharides (fructooligosaccharides, xylooligosaccharides, polydextrose and resistant dextrin), CRC patients showed increased serum levels of immunoglobulins (228). Also, higher level of beneficial bacteria like Bifidobacterium and Enterococcus were found in the perioperative period along with the increased abundance of *Escherichia-Shigella* in postoperative period (228). Pectin has exhibited anticancerous properties in various mice models and several cell lines, like Galactan rich pectin (RG-1) and modified citrus pectin inhibit cancer metastasis. Ohkami et. al, reported antitumor role of apple pectin in AOM induced colon carcinogenic rat (**Table 1**) (240).

**Table 1:** Probiotic bacterial composition and their anticancer mechanism.

S. No.	Pre/Pro/Syn/Post-Biotic composition	Anticancer mechanism	References
1	(Prebiotic) Fructooligosaccharides, xylooligosaccharides, polydextrose and resistant dextrin	Enhanced serum levels of IgG, IgM and transferrin.	(228)
2	(VSL#3) *Lactobacillus casei, L. plantarum, L. bulgaricus, and L. acidophilus, B. longum, B. breve and B. infantis*, and *Streptococcus thermophilus*	Upregulation of TNF-α.	(229)
3	(Prohep) *Lactobacillus rhamnosus* GG (LGG), *E. coli* strain Nissle 1917 (EcN), heat inactivated VSL#3.	Downregulation of IL-17	(230)
4	*Lactobacillus rhamnosus GG (LGG)*	Induction of type 1 interferon- β, Upregulation of pro-apoptotic proteins BAX, caspase-3 and p53. Downregulating the expression of β-catenin, COX-2, NFκB-p65 and Bcl-2.	(231, 232)
5	*Lactobacillus casei Shirota (LcS)*	Upregulation of IFN-γ, IL-1β and TNF-α.	(233)
6	*Lactobacillus acidophilus* La-14 SD-5212 and *Bifidobacterium longum* subsp. *Longum* 35624	Downregulation of COX-2.	(234)
7	*Bifidobacterium breve* and *Bifidobacterium longum*	Induction of tumor specific T- cells.	(235)
8	(Synbiotics) VSL#3^®^ (*Streptococcus thermophilus* BT01, *Bifidobacterium breve* BB02, *B. animalis* subsp. *Lactis* BI04, *L. acidophilus* BA05, *L. plantarum* BP06, *L. paracasei* BP07 and *L. helveticus* BD08 with prebiotic yacon based product (PBY)	Upregulation of IL-2 & IL-4	(236)
9	(Synbiotics) Raftilose synergy1^®^ with LGG and *B. lactis* Bb12	Reduction of GST-P enzyme	(237)
10	(Postbiotic) *L. plantarum* UL4 produced PM	Induction of apoptosis	(238)
11	(Postbiotic) Ferrichrome derived from *L. casei*	Increased DDIT3, Activation of caspase 3	(239)

### 5.2 Probiotics

According to the World Health Organization (WHO), probiotics are defined as ‘live microorganisms’ which when administered in adequate amount confer a healthy benefit on the host (241). Probiotics are widely used as standardized food supplements and recognized for their safety (242). Probiotics exert health benefits by the production of SCFAs, specifically butyrate, through the fermentation of polysaccharides by *Clostridium butyricum* and *Akkermansia muciniphila* (243). Butyrate exhibits wide-ranging effects, including the regulation of immune function, intestinal hormone production, and lipogenesis. Studies indicate that butyrate producing Firmicutes family plays a role in apoptosis by reducing cell proliferation. Additionally, *Bifidobacterium bifidum* increases butyrate production leading to apoptosis induction in malignant CRC cells (226, 244, 245). Beyond the well-established production of SCFAs, specific probiotic formulations, such as VSL#3 composed of eight different strains containing Bifidobacteria (three), Lactobacilli, (four) *and* Streptococcus (one), demonstrated the capability to synthesize conjugated linoleic acid (CLA). Interestingly, in an *in vivo* AOM-induced murine CRC model, VSL#3 administration resulted in the upregulation of colonic TNF-α compared to the untreated control group further suggesting a potential immunomodulatory role of VSL#3 linked to enhanced epithelial healing. Also, CLA has a role in inhibiting COX-2 expression and inducing apoptosis (229). A study by Li et al. investigated the therapeutic effects of a novel formulation of three strains designated as ‘Prohep’ further supports the potential of probiotics in HCC (230). ‘Prohep’ formulation includes *Lactobacillus rhamnosus* GG (LGG) as a keystone species present in the human gut, viable *E. coli* strain Nissle 1917 (EcN), and the immunomodulatory components of heat inactivated VSL#3. Notably, this study was performed in an *in vivo* model of HCC and demonstrated a significant tumor reduction within the treated mice by downregulating the interleukin 17 (IL-17) expression and further weakening the angiogenesis (**Figure 4**) (230). Studies also revealed a significant correlation between the abundance and diversity of intestinal flora and the effectiveness of PD-1 inhibitor treatment, ultimately impacting patient survival (246). LGG when administered orally or in combination with anti-PD-1 in melanoma and CRC-murine model, increases the anticancer function of dendritic cells (DC) via activated CD8+ T cells in the TME. This combination showed a significantly stronger effect to suppress tumor growth when given alongside immune checkpoint blockade (ICB) therapy. LGG triggers the production of Type 1 interferon- β (IFN- β) via cGAS/STING/TBK and interferon regulatory factor-7 (IRF-7) pathway (231).

Conversely, in colon cancer cells LGG treatment upregulated the expression of pro-apoptotic proteins Bax, caspase 3, and p53 (232). LGG cell-free supernatant (LGG-SN) demonstrated selective cytotoxicity towards HT-29, Caco-2 and HCT-116 colon cancer cell line and A375 metastatic melanoma cancer cells, inducing a G2/M cell cycle arrest and reducing the viability. Additionally, LGG-SN exhibits a synergistic effect when combined with chemotherapeutic agents like 5-Fluorouracil (5FU) and irinotecan, potentially enhancing their efficacy against cancer cells (247). *Lactobacillus casei Shirota* (LcS) has also been implicated in exhibiting anti-tumor properties. Studies suggest the potential efficacy of LcS against transplantable tumor models and chemically induced carcinogenesis. The underlying mechanism involves modulation of the host immune response, particularly via cellular arm enhancement. LcS administration has been shown to upregulate the production of cytokines such as IFN-γ, IL-1β, and TNF-α, which play a crucial role in activating immune effector cells to target and eliminate tumor cells (233). Furthermore, *in vitro* study using human gastric adenocarcinoma (AGS) and bladder (J253) cell lines demonstrated the ability of specific probiotic strains to induce apoptosis in cancer cells. *Lactobacillus acidophilus* La-14 (SD-5212) and *Bifidobacterium longum* subsp. Longum 35624 has been shown to induce morphological changes indicative of apoptosis suggesting a dose-dependent anti-proliferative effect (234). These same strains have also exhibited anti-angiogenic potential by downregulating COX-2 expression, an enzyme linked to tumorigenesis, blood vessel growth (angiogenesis), and cancer cell survival (234).

A study conducted by Sivan. et al. investigated the influence of gut microbiota on tumor growth employed genetically identical C57BL/6 mice harboring distinct gut microbial communities obtained from two sources: Jackson Laboratory (JAX) and Taconic Farms (TAC) (235). Notably, tumors grew significantly faster in TAC mice compared to JAX mice. This disparity correlated with a more robust tumor-specific T cell response and intratumoral accumulation of CD8+ T cells in JAX mice. To elucidate the potential immunomodulatory role of gut microbiota, researchers performed fecal microbiota transplantation (FMT) by orally administering fecal suspensions from JAX or TAC mice to TAC and JAX recipients before tumor implantation. FMT from JAX mice significantly delayed tumor growth in TAC recipients. Subsequent analysis revealed a strong association between *Bifidobacterium* abundance (400-fold higher in JAX-fed TAC mice) and the observed antitumor T cell response (235). This finding led to the investigation of a commercially available Bifidobacterium cocktail containing *B. breve* and *B. longum.* Oral administration of this cocktail, alone or combined with the immune checkpoint inhibitor (ICI) αPD-L1, to TAC mice with established tumors, resulted in significantly improved tumor control compared to untreated controls. This effect correlated with a robust induction of tumor-specific T cells in the periphery. Interestingly, the antitumor effect of Bifidobacterium extended beyond B16 melanoma, with similar observations in TAC mice bearing either B16 or MB49 bladder cancer cells. These findings suggest that *Bifidobacterium* may activate host dendritic cells, thereby enhancing the function of tumor specific CD8+ T cells, ultimately leading to improved tumor control (235).

Further, probiotics modulate the immune response by enhancing the intestinal barrier function through regulation of tight junction proteins, thereby promoting anti-inflammatory cytokine production, and potentially activating antigen-presenting cells (APCs) that prime tumor-specific T cells (248). This strengthened barrier prevents the translocation of harmful agents and dampens chronic inflammation, a known risk factor for cancer development (248). These studies highlight the complexity of probiotic-host interactions, emphasizing the need for further research to elucidate strain-specific effects and optimize probiotic interventions for cancer immunotherapy. Live biotherapeutic products (LBPs), the therapeutics based on probiotics and live bacteria, are developed to prevent, treat, or cure a human disease (242). Despite the numerous attempts by researchers, only *Bacillus Calmette-Guérin* (attenuated *Mycobacterium bovis*) has been approved for human use. Here, as an improved method, instead of live attenuated bacteria have been used to treat bladder cancer (249). In another ongoing human clinical study, the effects of a single strain LPB (*Christensenella minuta)* has been tested to treat obesity and other metabolic diseases (**Table 1**) (250).

### 5.3 Synbiotics

A mixture containing live microorganisms and substrate(s) that are selectively utilized by host microorganisms, confering health benefits on the host is known as synbiotics (251). A synbiotic containing probiotics VSL#3^®^ (*Streptococcus thermophilus* BT01, *B. breve* BB02, *B. animalis* subsp. *Lactis* BL03, *B. animalis* susp *lactis* BI04, *L. plantarum* BP06, *L. paracasei* BP07, *L. helveticus* BD08) and prebiotic yacon based product (PBY) modulated the composition of mice intestinal microbiota (**Figure 4**). The symbiotic group experienced 38.1% reduction in the incidence of pre-neoplastic lesions in mice colon as compared to the control group. Also, biotic group of mice had high concentrations of SCFA as compared to control and probiotic mice. Upregulation of IL-2 and IL-4 was observed in synbiotic group, which helps in improvement of immune response and antitumor defense (236). Raftilose Synergy 1^®^ composed of oligofructose enriched inulin and probiotics containing LGG and *B. lactis* Bb12, served as a synbiotic given to AOM induced colon cancer infected mice and significantly reduced tumors as compared to the control group. Also, significant reduction of Glutathione S-transferase placental enzyme pi type (GST-P) expression was observed (**Table 1**) (237).

### 5.4 Postbiotics

According to International Scientific Association of Probiotics and Prebiotics (ISAPP), postbiotics are described as preparation of inanimate microorganisms and/or their components that confers a health benefit on the host (252). The postbiotic metabolite (PM) produced by *L. plantarum* UL4 (**Figure 4**) demonstrated significant induction of apoptosis in human breast cancer cells MCF-7 (238). Also, after 48 hours cell cycle was arrested at G0/G1 phase. In AOM-dextran sulphate sodium (DSS)-induced carcinogenesis mouse model, ‘ferrichrome’ derived from *L. casei* ATCC 334, led to a reduction in tumor size. Ferrichrome increased the DNA damage inducible transcript 3 (DDIT3) signal and activated caspase-3 pathway, leading to DNA fragmentation. Also, ferichrome combined with 5FU significantly decreased the tumor size (**Table 1**) (239).

## 6 Targeting the gut microbiome for improved cancer therapy

The correlation between antibody treatment and reduced gut population has been observed in melanoma, and colon cancer. In melanoma (B16) and colon (MC38) mouse models, antibody administration has been reported to imperial the immunotherapy efficacy and reduce the inflammatory cytokine production using anti-IL-10/CpG oligodeoxynucleotides (253). Clinical data indicates that systemic use of antibiotics results in less effective ICI (254). In laboratory mice with MC-26 carcinoma cell implants, the efficacy of gemcitabine was found to be restored when it was directed against intratumoral bacteria (255). On the other hand, it has also been established that broad-spectrum antibiotic treatments have negative impacts on tumor therapy. In patients receiving antibiotics for non-small cell lung cancer (NSCLC) had lower survival rates both before and after the treatment, while patients receiving ICIs for advanced melanomas and allogenic hematopoietic cell transfer therapy reported evidence of antibiotic mediated loss of *Bifidobacterium* spp. or *Akkermansia spp* (256). Similarly, the negative effect of antibiotics in patients with hematologic malignancies was studied and the overall reduced survival rate was observed in patients receiving anti-Gram-positive antibiotics. Thus, methods are being developed to reduce the impact of antibiotics on the native microbiota. The use of ‘tailored microbiome therapy’ may help increase therapeutic response by selectively reducing the microbial communities. Current research focuses on developing new strategies that allow for the targeted removal of commensals that promote cancer while having little effect on the microbiome.

Bacteriophages have the ability to directly destroy bacteria and are known to target tumor microbiome and modulate immunity, making them the ideal treatment option for clinical applications (257). Systemic administration of three Myoviridae bacteriophages in 13 patients infected with *S. aureus* infection displayed preliminary effectiveness (258). It has been reported that use of phage preparations enables the targeted suppression of cancer promoting commensals, while having low impact on surrounding microbiome. For example, in the AGS human gastric adenocarcinoma cell line, *H. pylori, a* lytic bacteriophage demonstrated the synergistic activity of cancer-associated commensal. Further, *H. pylori* infected AGS cells showed reduced reactive oxygen species (ROS) accumulation when treated with Hp phage (259). According to Gogokhia et al., dsDNA from Caudovirales phages activates the innate immune response by interacting with TLR9. This shows that phages reduce the colitis and intestinal inflammation. Further, they also reduce colon cancer caused by bacteria and hence benefit the host immune system by stimulating TLR9 (260).

**Figure 5:**
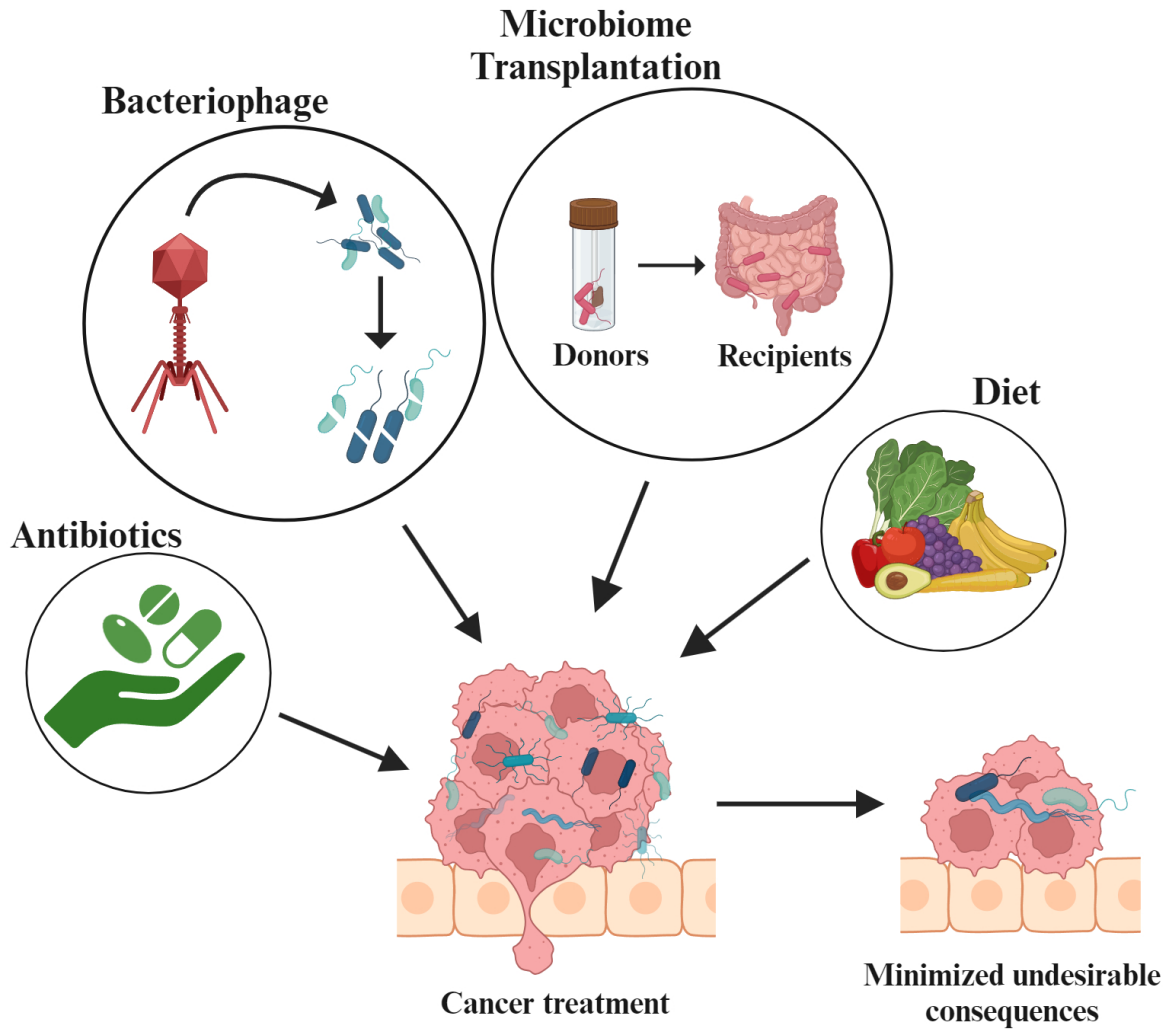
Utilizing gut microbiota-based strategies for cancer management.

Dietary modifications and microbiome transplantation are also known to regulate the microbiome alteration that aids in cancer treatment. Dietary modification has a major influence on gut microbiome. For example, animal fat removal demonstrated a significant reduction in Bacteroidales (261) whereas a high-fiber diet consumption led to an increase in SCFAs-producing bacteria upon consuming a high-fiber diet. Such shifts to high-fiber diet, exhibited pro-apoptotic and anti-inflammatory responses in colorectal mouse cancer models (262). Similarly, it was observed that inulin administration in adenocarcinoma mouse models improved the effectiveness of anti-PD-1 therapy while simultaneously increasing the relative abundance of Roseburia, Lactobacillus, and Akkermansia (263). Microbiome transplantation has been widely accepted as a therapy where disease-associated microbiome is replaced with a healthy individual through FMT. Studies have supported the enhanced immnotherapy via transferring the patient fecal sample into antibiotic treated specific-pathogen free (SPF) mice (264). Microbes, in conjunction with ICIs or chemotherapy regimens can also be adopted in the treatment of cancer patients (265). Additionally, human clinical trials highlighted that in a fraction of FMT recipients, particularly melanoma patients who earlier responded to ICI treatment but later developed ICI-resistance, FMT restored the ICI non-responsiveness (266, 267). Thus, targeting the gut microbiome may represent a novel approach for cancer prevention and treatment (**Figure 5**).

## 7 Discussion

The interplay of the gut microbiome and dietary micronutrients holds a captivating avenue for exploring potential cancer prevention strategies and treatment. In this review, we delved into the intricate mechanisms and contributions of micronutrients and gut bacteria in cancer treatment. Micronutrients have specific characteristics like anti-inflammatory and antioxidant properties and modulate the gene expression with impact on cell proliferation and apoptosis. Adequate intake of vitamin B12, folic acid, vitamin D, selenium, and carotenoids have been reported to significantly reduce the risk of cancer. The human gut microbiome plays a pivotal role in nutrient metabolism, immune system development, and drug metabolism. Disruptions in dietary patterns and gut microbiome are linked to cancer progression. A fiber-rich diet promotes the growth of healthy bacteria like Firmicutes which leads to the production of SCFA with enhanced anticancer properties. Vitamin B complex exhibits prebiotic potential that influences beneficial bacterial growth. Similarly, minerals like zinc and selenium play a crucial role in gut health and influence cancer therapies. The tumor immune microenvironment plays a crucial role in cancer prognosis and treatment process, with recent studies indicating that the lung microbiome may influence immune cell behaviour and cancer progression (268). Studies reveal a strong correlation between the composition of gut microbiome and patient survival. For example, *F. nucleatum*, significantly abundant in CRC, promotes cell invasion and metastasis (148). Similarly, *H. pylori* infection, a major risk factor for gastric cancer, disrupts cellular pathways and actively involved in cell cycle regulation (159). Furthermore, particular strains of *B. fragilis*, highly enriched in CRC patients, contribute to impaired tissue integrity, chronic inflammation, and dysfunctional immune response, thereby promoting tumor growth and development (179). Additionally, pathogenic *E. coli* strains can induce inflammation, oxidative stress, and DNA damage in host cells. They can also manipulate the cell cycle, hence leading to tumor formation (182). Interestingly, even *P. acnes*, typically associated with acne, has been linked to prostate cancer, with certain strains triggering inflammation and promoting cell proliferation (201). However, the crosstalk between bacteria and cancer cells not always results in tumor development. For instance, bacteria such as Pseudomonas aeruginosa, secretes azurin in the presence of cancer cells, with a dose-dependent relationship and enhances patient survival in breast and melanoma cancer cases (269). Thus, understanding the distinct roles of cancer-associated bacteria in carcinogenesis can lead to the development of innovative treatments and strategies. The oral microbiome, alongside the gut microbiome, holds promise as a diagnostic and prognostic tool for various cancers and serve as a therapeutic target (270). Natural polyphenols have emerged as an effective cancer treatment therapy with their antioxidant-like properties (271). Prebiotics, probiotics, synbiotics and postbiotics exert beneficial effects through multiple mechanisms. One key mechanism involves the production of SCFAs, particularly butyrate, during dietary fiber fermentation by probiotic bacteria like *C. butyricum* and *A. muciniphila*. Butyrate-producing Firmicutes families are reported to induce apoptosis in cancer cells. Other probiotic formulations like VSL#3, synthesizes CLA and exhibits potential immunomodulatory effects. Furthermore, ‘Prohep’ demonstrates a significant reduction in tumor burden in HCC mice model. Recent findings highlight a critical link between the gut microbiome and the efficacy of PD-1/PD-L1 checkpoint inhibitor immunotherapy (246). The genetic modulation of gut microbiome using targeted dietary interventions or by probiotic supplementation and simultaneously optimized micronutrient intake may enhance the efficacy of the cancer treatment. By strategic modulation of the gut microbiome and targeted suppression of detrimental bacterial populations, while upregulation of beneficial bacteria, significant advancement can be achieved in cancer prevention.

## 8 Future perspective

Understanding, the gut bacteria influence on cancer metabolism and immune response is critical for developing targeted therapies. By deciphering these molecular pathways, novel therapeutic strategies can be unlocked. This requires isolation and characterization of specific probiotic strains with demonstrably positive effects on the gut microbiome composition and anti-tumor properties. Probiotic-based therapies can offer promising avenues for the treatment of cancer. Genetically engineered probiotics are capable of modulating immune responses against tumor cells by producing tumor-killing agents, which may offer enhanced efficacy and reduced collateral damage. The synergistic combination of prebiotics and probiotics can provide a nutrient-rich environment that supports beneficial bacteria, potentially optimizing treatment outcomes. Further, personalized probiotic therapy may facilitate the selection of the optimal and most effective probiotic strains for specific tumor types. Prebiotic interventions offer significant promise. By selectively promoting the growth of beneficial bacteria, prebiotics can contribute to a balanced microbiome required to foster a robust immune response and reduce inflammation. These factors are crucial in combating cancer, as a strong immune system and reduced inflammatory environment can help inhibit tumor growth and progression. Thus, the future of cancer therapy holds immense potential for leveraging the power of the gut microbiome. By carefully selecting and administering probiotics and prebiotics, we may be able to develop targeted therapies against tumors while safeguarding the health of vital organ systems. As our understanding of the microbiome-cancer axis continues to evolve, we can anticipate exciting advancements in personalized medicine and therapy. Further, meticulous characterization of these strains can pave the way for personalized cancer treatment strategies tailored to an individual’s unique gut microbiome. However, establishing the efficacy and safety of probiotic interventions is crucial. Well-designed clinical trials are essential for evaluating the potential of probiotic supplementation as an adjunct therapy in cancer prevention and treatment, as well as in managing side effects associated with chemotherapy. Thus, by pursuing this multifaceted approach, we can harness the power of the gut microbiome to revolutionize cancer care.
